# Are we joining the One Health dots? A scoping review of research on the one health effects of extreme weather events in eastern Australia

**DOI:** 10.3389/fvets.2024.1423501

**Published:** 2024-07-29

**Authors:** Rebecca Ward, Victoria J. Brookes, Kazi Mizanur Rahman

**Affiliations:** ^1^The University of Sydney, University Centre for Rural Health, Lismore, NSW, Australia; ^2^Sydney Medical School, The University of Sydney, Camperdown, NSW, Australia; ^3^Sydney School of Veterinary Science, Faculty of Science, The University of Sydney, Camperdown, NSW, Australia; ^4^Sydney Infectious Diseases Institute, Faculty of Medicine and Health, The University of Sydney, Camperdown, NSW, Australia; ^5^Faculty of Health Sciences and Medicine, Bond University, Robina, QLD, Australia

**Keywords:** extreme weather events, human health, animal health, ecosystems, One Health, eastern Australia, scoping review

## Abstract

Extreme weather events such as floods, bushfires, cyclones, and drought, are projected to increase in eastern Australia. Understanding how these events influence the combined, sustainable well-being of humans, animals, and ecosystems – that is One Health – will enable development of transdisciplinary and ultimately more effective interventions. A scoping review was conducted to explore the research associated with the effects of extreme weather events in eastern Australia using a One Health lens, specifically identifying the type of extreme weather events studied, the research conducted in the context of One Health, and gaps to inform improved One Health implementation. The review followed JBI guidelines (based on PRISMA). Eligible research was peer-reviewed, in English, and published since 2007, in which primary research studies investigated the impact of extreme weather events in eastern Australia on at least two of ecosystems, human health, and animal health. Using structured search terms, six databases were searched. Following removal of duplicates, 870 records were screened by two reviewers. Eleven records were eligible for data extraction and charting. The scope of extreme weather events studied was relatively limited, with studies in flood and bushfire settings predominating, but relatively little research on cyclones. Major health themes included more than the impact of extreme weather events on physical health (zoonotic and vector-borne diseases) through investigation of social well-being and mental health in the context of the human-animal bond in evacuation behaviors and drought. Research gaps include studies across a broader range of extreme weather events and health topics, as well as a more comprehensive approach to including the impacts of extreme weather events on all three domains of One Health. The limited research focus inevitably translates to limited recommendations for policy, planning and response to manage extreme weather event emergencies. Given the expected increase in frequency of these events, there is a critical need for more comprehensive primary research to better identify strategies and facilitate implementation of One Health promotion for improved outcomes in extreme weather event emergencies.

## 1 Introduction

In March 2023, the Intergovernmental Panel on Climate Change (IPCC), a United Nations body to assess science in the context of climate change, published its Sixth Assessment Report (Synthesis Report), stating that global warming is projected to increase between 2021 and 2040 in nearly all modelled pathways (1). With further warming, extreme weather events will increase in every region globally (1).

Given the projected increase in frequency of extreme weather events, understanding how they influence the combined, sustainable well-being of humans, animals, and ecosystems – that is One Health – would enable development of transdisciplinary and ultimately more effective interventions. One Health, as defined by the One Health High-Level Expert Panel, is an integrated, unifying approach that aims to sustainably balance and optimize the health of people, animals, and ecosystems (2). It recognizes that humans, animals, and the ecosystem are interdependent, and as such encourages multiple sectors and disciplines to work collaboratively, at local, national, regional, and global levels. It has been suggested that an integrated One Health approach would be more effective in addressing the impact of climate change outcomes such as extreme weather events when compared to ecosystem, public health and animal health sectors working separately (3).

The impacts of extreme weather events have been demonstrated in the context of each of ecosystem, animal, and human health. For example, a systematic review found that flooding events on beef and pig farms in the United States of reduced affected productivity and biosecurity (4). In a study of an outbreak of Rift Valley fever in Kenya in 2006–2007, heavy rains and flooding led to the emergence of a large number of competent vectors, likely increasing the transmission of the virus to people (5). A systematic review of health outcomes after floods in sub-Saharan Africa supported this, finding that floods created breeding habitats for disease vectors, particularly mosquitoes, resulting in their population growth (6). In a study in Ethiopia, nationwide droughts in 2009 and 2015 caused widespread crop and livestock losses due to water scarcity and disease (7). These impacts have downstream health effects; the loss of agricultural productivity increased susceptibility to food insecurity and reduced spending on non-food expenses such as healthcare and increased the likelihood of stunting in children in this setting (7). There is also research on the impacts of extreme weather events on local natural ecosystems. For example, floods, cyclones and wildfires have all been demonstrated to detrimentally affect riverine ecosystems in Australia (8). A One Health approach in which all three domains are investigated and supported might significantly contribute to improved health in many areas by not only responding to downstream impacts such as emerging infectious diseases, increased antimicrobial resistance, and malnutrition, but also working to prevent health problems by addressing upstream drivers of health including food security, safe water systems, and prevention of spillover of pathogens that could result in emerging infectious disease.

**Figure 1:**
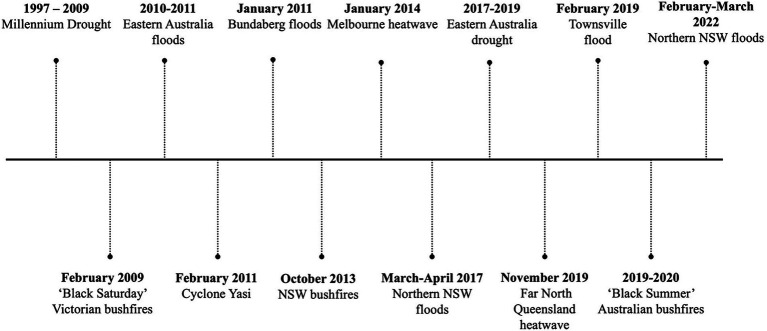
Timeline of major extreme weather events in eastern Australia from 1997 to 2022.

In Australia, the 2020 Royal Commission into National Natural Disaster Arrangements Report echoed the concerns of the IPCC report, with extreme rainfall and associated flooding, intensity of tropical cyclones and the frequency and severity of heatwaves and wildfires projected to increase within national borders in coming decades (9). Some of the most significant extreme weather events in eastern Australia in recent years include extreme droughts and wildfires. Notable examples include the severe drought that affected much of New South Wales (NSW) and Queensland (Qld) in 2017–2019 which culminated in the “Black Summer bushfires” in 2019–2020 (10). This occurred after the decade-long Millennium Drought from 1997 to 2009, in which there was widespread crop failure, loss of livestock, dust storms, and major wildfires across southeast Australia (11). Flash and riverine flooding significantly affected areas of northern NSW and Qld in 2017 and again in February–March 2022, damaging >20,000 properties (12, 13). Additionally, several tropical cyclones have impacted North Qld, most notably tropical cyclone Yasi in 2011 and tropical cyclone Marcia in 2015 (14, 15). A timeline of key extreme weather event in eastern Australia is outlined in Figure 1, in which human impacts (mortality, injuries, evacuation rates and property damage) were recorded by government agencies (16).

The objective of this review was to determine the frequency and contexts of One Health approaches in association with research on extreme weather events in eastern Australia. We aimed to provide a baseline of current approaches and contexts to inform future responses. Specifically, the review investigated the nature of extreme weather events that have been investigated, the impacts experienced across ecosystem, human and animal health, the mitigation measures that were implemented, and recommendations. We also identified gaps in the current approach to extreme weather events from a One Health perspective.

## 2 Methods

This study was conducted following JBI guidelines on scoping reviews (17). Reporting was guided by the Preferred Reporting Items for Systematic Reviews and Meta-Analyses Extension for Scoping Reviews (PRISMA-ScR) checklist (18).

### 2.1 Eligibility criteria

#### 2.1.1 Population of interest

The population of interest was living organisms in the domains of humans, animals, and ecosystems, impacted by extreme weather events. Ecosystem refers to “a geographic area where plants, animals, and other organisms, as well as weather and landscapes, work together to form a bubble of life” (19). Therefore, in our review, wild animals were considered part of an ecosystem and ‘animal’ refers to companion animals, livestock, and other animals that are maintained directly by humans (for example, animals in zoological collections or research facilities).

#### 2.1.2 Concept

The concept of this review was the impact of extreme weather events on ecosystems, human health, and animal health. Due to the paucity of information integrating all three domains of One Health recognized during initial literature searching, studies examining any two of the three domains were included. The specific extreme weather events examined include floods, wildfires, droughts, tropical cyclones, heatwaves, landslides, and tidal waves. In this review, human health considers physical, mental and social well-being, as defined by the World Health Organization: “health is a state of complete physical, mental and social well-being and not merely the absence of disease or infirmity” (20).

#### 2.1.3 Context

The context was confined geographically to mainland eastern Australia, including the states of Queensland (Qld), New South Wales (NSW), and Victoria, and the Australian Capital Territory (ACT).

#### 2.1.4 Information sources

Inclusion and exclusion criteria are described in Table 1. Records that reported primary research with quantitative, qualitative, or mixed methods designs conducted in the study region were included. Gray literature, including government, industry and NGO reports were not included. Only studies published in English were included. Studies that examined the impact of climate change without specific extreme weather events were excluded.

**Table 1:** Inclusion and exclusion criteria.

Inclusion criteria	Exclusion criteria
Population – humans, animals and ecosystems affected by extreme weather events including floods, tidal waves, landslides, droughts, bushfires, heatwaves and cyclones.	Does note meet inclusion criteria for population.
Concept – has One health specific focus or examines health of humans and ecosystems, humans and animals or animals and ecosystems	Does not meet inclusion criteria for concept, that is, relates to only one of human health, animal health, or ecosystems.
Context – confined to Eastern Australia, including the states of Queensland, New South Wales, Victoria and Australian Capital Territory.	Does not meet inclusion criteria for context, that is, is outside of eastern Australia.
Information sources – published studies of primary research with quantitative, qualitative, or mixed-method designs. Published in or after 2007. Published in English.	Does not present primary research findings.
	Published before 2007.
	Examines extreme weather events occurring only before 2007.
	Publication not in English.
	Full text not available.

### 2.2 Search

Following an initial limited search of Web of Science and Scopus to identify relevant records and key words, a comprehensive search strategy was developed in consultation with an academic librarian to identify eligible records. Searches were restricted to studies published in English, and since 2007. This year was chosen to coincide with the formation of the One Health Tripartite and publication of, ‘Contributing to One World, One Health. A Strategic Framework for Reducing Risks of Infectious Diseases at the Animal-Human-Ecosystems Interface’ (21). Databases searched include MEDLINE (Table 2), CINAHL, PROQUEST, Scopus, Web of Science and Informit (database links and search strings for each database are included in the Supplementary material). A search of all databases was conducted on 16 June 2023.

**Table 2:** Search terms used for Medline database.

Key Concept	MeSH Terms and Keywords
One Health	One Health OR one health*.mp OR one medicine*.mp OR planetary health*.mp OR ecohealth*.mp OR ((human* or “public health*” or “environmental health*”) and (Animal* or wildlife* or livestock*)).mp OR ((human* or “public health” or “environmental health”) and (ecosystem* or ecolog*)).mp OR ((Animal* or wildlife* or livestock*) and (ecosystem* or ecolog*)).mp
Extreme weather events	exp natural disasters/ OR cyclonic storms/ OR droughts/ OR floods/ OR landslides/ OR tornadoes/ OR wildfires/ OR Extreme Weather/ or Extreme Cold Weather/ or Extreme Hot Weather/ OR (natural disaster* OR cyclon* OR drought* OR flood* OR landslide* OR tidal wave* OR tornado* OR wildfire* OR bushfire* OR peat fire* OR landscape fire* OR extreme heat* OR landslide* OR heat wave* OR Extreme weather* OR extreme cold weather* or extreme hot weather*).mp
Eastern Australia	australian capital territory/ or new south wales/ or queensland/ or victoria/ (australian capital territory* or new south wales* or queensland* or victoria* or east* australia*).mp

### 2.3 Selection of sources of evidence

Following the systematic searches, all identified records were collated and uploaded to EndNote 20 (22), a reference management tool, and Covidence (23), a web-based platform to support systematic reviews. Duplicate records were removed. Record titles and abstracts were screened by the lead reviewer (RW) and potentially relevant records were retrieved in full and assessed by two reviewers (RW and either KR or VB). Conflicts during both title and abstract screening, and full-text review were discussed to reach consensus.

### 2.4 Data charting

Data were extracted by one reviewer (RW) using an *a priori* data extraction tool developed in Covidence after discussion with all the reviewers (Supplementary material). Data were reviewed by a second reviewer (KR or VB). Conflicts were discussed to reach consensus. The extracted data included details about the study type, population, site, time period, type of extreme weather event, domains of One Health examined, key findings and recommendations in the context of One Health.

### 2.5 Analysis and presentation of results

Tabular, graphic, and narrative methods were used to present the data extracted from eligible studies. There was no bias assessment of individual records, as is typical of scoping reviews.

## 3 Results

### 3.1 Search result and study selection

**Figure 2:**
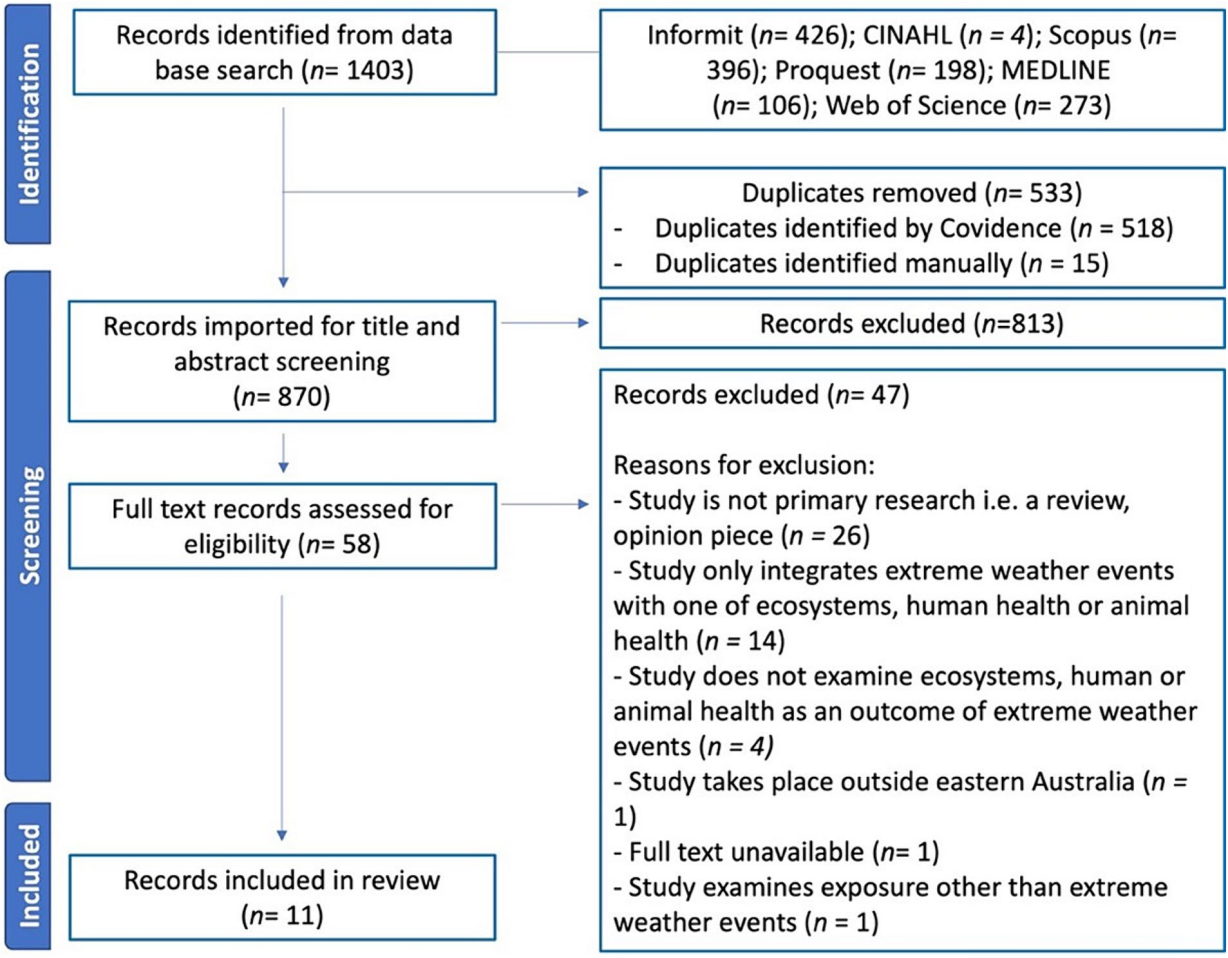
Preferred reporting items for systematic reviews and meta-analyses extension for scoping reviews (PRISMA-ScR) flow chart showing the number of records included or excluded at each stage of screening.

Figure 2 shows a PRISMA flow chart of articles included and excluded in each stage of identification and screening. The database searches identified 1,403 records. After removal of duplicates, 870 records were screened on their title and abstract for eligibility. Eight hundred and twelve records were excluded, leaving 58 records for full-text screening. Common reasons for exclusion at this stage were that records did not include an extreme weather event or did not take place in eastern Australia. During full text screening, 47 records were excluded. The most common reasons for exclusion at this stage were that the study was not primary research (55%, *n* = 26) or that the study only explored one domain of One Health, that is, either human health, animal health or ecosystems (30%, *n* = 14). Eleven articles were eligible for data extraction.

### 3.2 Inclusion of sources of evidence

Table 3 provides a summary of the characteristics of the 11 eligible records. Most studies included data from NSW (*n =* 8) or Queensland (*n* = 6), with four studies including data from multiple states or territories in eastern Australia. Only one study was conducted in Victoria alone. Eligible records used quantitative only (*n* = 5), qualitative only (*n* = 3), and mixed methods (*n* = 3) research. Qualitative data was most commonly collected through interviews (*n* = 3). Included sources comprised records spanning 2014 to 2022, with the highest number in 2020 (*n* = 3), followed by 2014 (*n* = 2) and 2019 (*n = 2*).

**Table 3:** Charted data from eligible studies.

First author, year, reference	Study population	Location (state)	Study design	Extreme weather event	Domains of One Health investigated	Main findings
Adekunle, 2019 (24)	Human cases of mosquito borne disease (MBD)	Queensland	Quantitative	Flood	Human health; ecosystems	A flooding event is likely to increase the number of mosquito-borne infection and increase the carrying capacity of the vector population. After an initial washing out event, it is expected that there will be an addition number of breeding sites due to flooding. Using the 2019 year-to-date number of notifiable MBDs, an increase in the number of MBD cases was modelled, with a peak at 104, one-half month after the flood. Flooding can also displace animal populations such as kangaroos and wallabies, reservoirs of Ross River virus, and lead to transmission to humans.
Baranowski, 2021 (25)	Grey-headed flying foxes (*Pteropus poliocephalus*)	Queensland, New South Wales, Australian Capital Territory, Victoria	Quantitative	Bushfire	Human health; ecosystems	Bushfire leads to the drastic reduction of available foraging resources for the grey-headed flying fox, the most important of which being key winter resources. Responses to such events include migration of animals to unaffected areas. Due to deforestation, a significant proportion of alternate grey-headed flying fox habitat has been lost, forcing animals to roost in urban areas, which increased risk of spillover of Australian bat lyssavirus and Hendra virus into humans.
McCarthy, 2018 (26)	Residents who own, care for or work with animals	New South Wales	Qualitative; quantitative	Bushfire	Human health; animal health	The strength of the human-animal bond influences decision-making during emergencies. Most people ‘definitely would not’ or ‘might not’ evacuate if advised by authorities if they could not bring their pets. Many respondents (53%) did not have a clear plan of how to evacuate with their pets. Specific concerns about animal behavior were mentioned that would increase the stress and time to evacuate. Most respondents were not aware of where to seek information about what to do with their animals during and emergency.
Merone, 2020 (27)	Bats (*Pteropus conspicillatus*)	Queensland	Qualitative	Heatwave	Human health; ecosystems	The heatwave caused the mass mortality of more than 4,000 bats. This was associated with increased human-bat contact and risk of transmission of Australian bat lyssavirus, increased emergency department presentations due to injury through handling bats, increased risk of needle stick injury from untrained members of the public trying to administer intra-venous rehydration and health-service strain. Psychological distress associated with living with pungent malodour and the sight of mass-animal death was reported
Perceval, 2019 (28)	Australian farmers	Queensland, New South Wales	Qualitative	Drought	Human health; animal health	Death and suffering of livestock during drought adversely affect the mental health of farmers. Participants expressed distress in watching their animals suffer, and the difficulty with having to euthanize them.
Prow, 2014 (29)	Humans and feral rabbits	New South Wales	Quantitative	Flood	Human health; animal health; ecosystems	Following extensive flooding and subsequent ideal conditions for mosquito breeding, an outbreak of equine encephalitis occurred, leading to the isolation of the first virulent strain of West Nile virus (WNV) in Australia. Humans appear to not have been exposed to WNV during the equine epidemic, with no significant difference between pre- and post-2011 serological data. As such, humans may still be vulnerable to this virulent strain of WNV as they do not have prior exposure. It remains to be determined whether wild rabbits can develop a high enough viraemia to contribute to WNV transmission.
Sanusi, 2020 (30)	*Platanus acerifolia* (London Plane tree)	Victoria	Quantitative	Heatwave	Human health; ecosystems	Heatwave pushed *P. acerifolia* beyond its ambient temperature threshold and caused trees to drop a large proportion of their canopy. This resulted in a significantly reduced physiological equivalent temperature (an estimate human thermal comfort) difference between open areas and under tree canopies on subsequent hot days.
Selvey, 2014 (31)	Human cases of Murray Valley Encephalitis (MVE)	Queensland, New South Wales, Australian Capital Territory, Victoria	Mixed methods	Flood	Human health; ecosystems	MVE virus activity in south-eastern Australia followed the extensive rainfall and flooding in the Murray-Darling basic and adjacent areas. Flooding was associated with an increased in mosquito vector *Culex annulirostris.* There were 17 human cases reported. Most cases were linked to ‘high risk’ mosquito exposure activities such as fishing at dusk, camping near rivers, outdoor evening sport. Despite widespread sentinel chicken surveillance and cases in horses, only one case was reported in Victoria.
Tall, 2020 (32)	Human cases of Ross River virus disease	New South Wales	Quantitative	Flood	Human health; ecosystems	Flooding positively predicted outbreaks of Ross River virus disease in three of the eight climate zones studied. Why flood-predicted outbreaks occurred in only these regions is uncertain. Rainfall, irrigation, and population density was variable across the positively associated regions, and average annual temperatures similar across the positive regions and some of the negative regions, and climate conditions across the negatively associated zones was variable. Flooding promotes clustering of macropods (a potential reservoir) on dryland and may increase vector-host contact.
Taylor, 2015 (33)	Pet owners	Queensland, New South Wales, Australian Capital Territory, Victoria	Qualitative; quantitative	Flood; Bushfire; Cyclone; Other	Human health; animal health	Pets influence decision-making and the process of evacuation in an emergency event. Pets influence whether people evacuate, where they evacuate to, increase the stress of transportation, determine the mode of transportation used, slowed down the speed of evacuation and increased the number of trips needed to the evacuation site.
Travers, 2022 (34)	Emergency responders	New South Wales	Qualitative	Flood; Bushfire	Human health; animal health	Pet owners try to enter the hazard zone or ignore warning in attempt to rescue their pets, potentially placing their and emergency responders lives at risk. Emergency responders do not always have the capacity to save people’s pets, which can exact an emotional toll of responders. Animal rescue is only possible when there is enough capacity.

### 3.3 Review findings

#### 3.3.1 Extreme weather events

**Figure 3:**
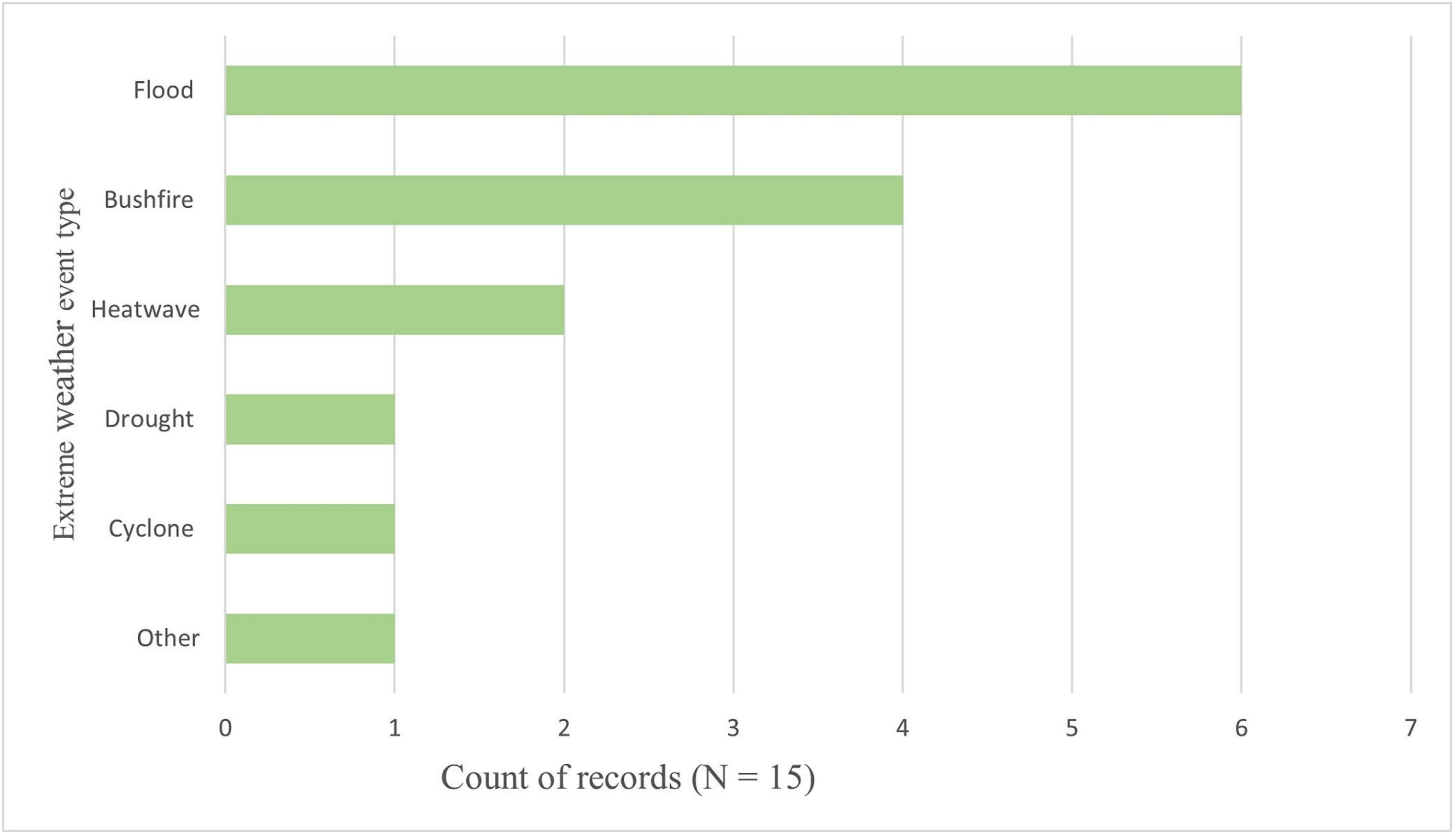
Count of extreme weather event types in eligible records.

Nine records included a single type of extreme weather event, while two included multiple types within a single study. The most frequent extreme weather event was flooding (*n* = 6) followed by wildfire (*n* = 4). Two studies investigated the impact of heatwaves, one investigated drought, and one investigated cyclone. No included studies included tidal waves or landslides. These findings are shown in Figure 3.

#### 3.3.2 Domains of One Health explored

**Figure 4:**
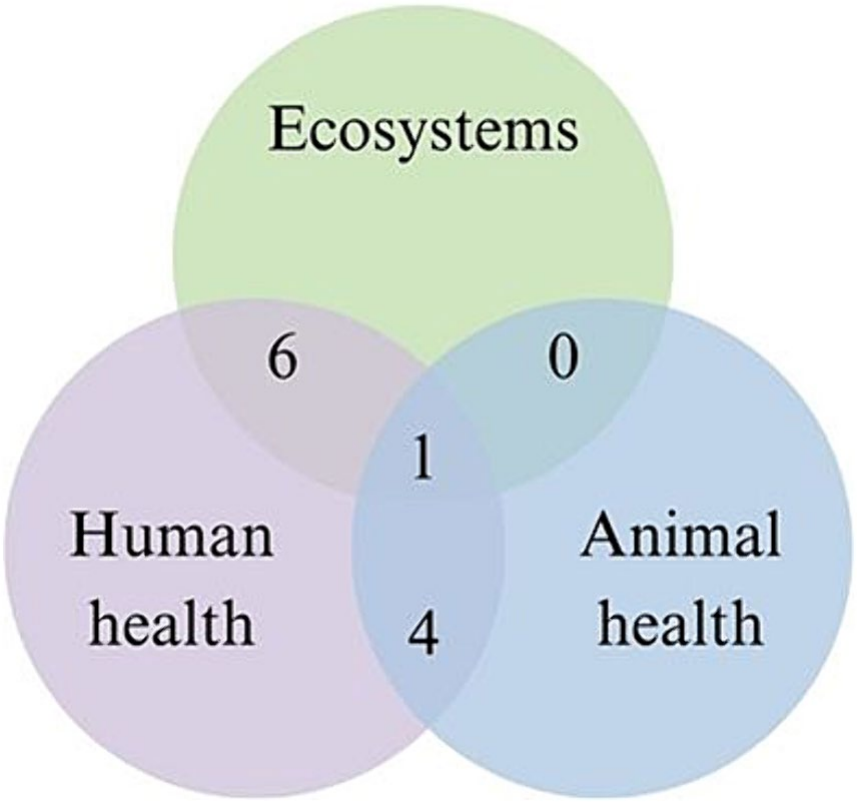
Venn diagram showing the number of domains of One Health investigated together.

Figure 4 shows the number of studies that include pairs or all three domains of One Health. The most commonly explored pair was human health and ecosystems (*n* = 6), followed by human health and animal health (*n* = 4). No studies integrated animal health and ecosystems. Only one study explored all three components of ecosystems, human health, and animal health. A variety of ecosystems were studied in the seven records that explored ecosystems; two were conducted exclusively in urban ecosystems, while the others were conducted in rural ecosystems (*n* = 3), or at the transition from a rural or ‘wild’ ecosystem to an urban ecosystem (*n* = 2).

#### 3.3.3 Zoonotic and vector-borne diseases

The relationship between extreme weather events and zoonotic and vector-borne diseases was a prominent theme in this review (6 of the 11 records). Of these, most (*n* = 5) focused on the impact of extreme weather events on ecosystems and human health, and the other incorporated all three domains of ecosystem, human health, and animal health.

Regarding extreme weather event type, four records were related to flooding, one related to heatwaves, and one related to wildfire. The diseases investigated were Australian bat lyssavirus (ABLV; *n* = 2), West Nile virus (WNV; *n =* 1), Murray Valley virus (*n* = 1), Ross River virus (*n* = 2), Barmah Forest virus (*n* = 1) and Hendra virus (*n =* 1). The broad findings were that extreme weather events increased the risk of disease. More specifically, flooding resulted in an increase in breeding sites for mosquito vectors, subsequently increasing risk of mosquito borne diseases (24, 29, 31). However, results were variable in a long-term quantitative study of Ross River virus disease outbreaks in inland NSW, where flooding was only associated with increased disease in certain regions, the reasons for which were unknown (32).

The study that included all three domains of One Health investigated the relationship between equine, feral rabbit and human WNV infection following flooding. It concluded that an equine outbreak of the disease was associated with flooding due to favorable mosquito breeding conditions. Humans appeared to not be infected with WNV during the outbreak period and could be vulnerable to future virulent strains, and it is possible that feral rabbits might be reservoirs for the virus (29). In another study, wildfire resulted in the loss of key winter resources for the grey-headed flying fox (*Pteropus poliocephalus* - a reservoir species for Hendra virus and ABLV), which could cause increased dispersal and fragmentation of populations over larger geographic areas, including into urban settings and increase risk of transmission of these viruses to humans (25). Flying foxes (spectacled flying foxes, *Pteropus conspicillatus* – also a reservoir species for ABLV) were also investigated in association with a heatwave in Far North Queensland which resulted in their migration and death in an urban area. This resulted in increased presentations by people to hospital for post-exposure prophylaxis due to direct contact with unwell or dead bats. Other human health impacts that were reported included increased needle-stick injury events due to untrained flying fox carers attempting to give intravenous rehydration therapy to unwell bats, and the psychological impact of living with pungent malodor associated with the site of mass-animal death (27). Of the included records examining zoonotic or vector-borne diseases, this latter study was the only study that made recommendations from a One Health perspective. Authors recommended the development of teams within public health units dedicated to assessing and preparing for climate-related risks, and partnering with other agencies to incorporate mass mortality plans into response protocols (27).

#### 3.3.4 Human-animal bond

The relationship between animals and their owners in the setting of an extreme weather event was explored by four records in this review. Three records examined the relationships between people and their pets, while one considered owners’ relationships with livestock.

Studies involving pets showed that the strength of the human-animal bond influences people’s decision-making during emergencies. Pets influenced many owners’ decisions about evacuation in the event of an extreme weather emergency, with sentiments such as “I would rather burn to death than leave my cats” (26). Most pet owners, when confronted with real or hypothetical extreme weather events requiring evacuation, stated that they “definitely would not” (43%) or “might not” (42%) evacuate if advised by authorities but were unable to evacuate with their pets (26). Pets also influenced where respondents evacuated to, the type of transportation used and increased the stress of evacuation (33). Notably, having pets slowed down the speed of evacuation and increased the number of trips needed to and from the evacuation site, increasing the risk of harm to their human carers and families (33). In a qualitative study of emergency responders involved in flood and wildfire responses, it was noted that pet owners are more likely to enter a hazard zone or ignore warnings in an attempt to save their pets, potentially placing both their own lives, as well as the lives of emergency responders, at risk. Additionally, when responders do not have the capacity to rescue pets, it was found that this imposes a significant mental and emotional burden on the responders themselves (34).

The death and suffering of livestock during severe droughts was reported as a significant contributor to poor mental health in farmers in NSW and Qld. Participants from all respondent groups, including NSW where drought conditions were reportedly less severe, spoke of their distress at witnessing the suffering of their livestock, and the emotional difficulty associated with conducting euthanasia (28).

Among the records that recognized the significance of human-animal bond in the setting of extreme weather events, only one provided recommendations from a One Health perspective (34). Authors recommended improvements to disaster management by addressing requirements for pets, engaging owners and community, and reorienting health and emergency services towards pet’s needs in disaster management practice, policy, and capacity building.

#### 3.3.5 Urban ecosystems change and human health

The final record examined how a heatwave altered an urban ecosystem and the effect on human health (30). In this Melbourne study, researchers found that London plane trees (*Plananus x acerifolia*), which are frequently planted in urban areas to provide shade and are perceived to be resilient to harsh conditions, are susceptible to sustained high temperatures, resulting in significant canopy loss. As a result, the street micro-meteorological conditions are altered, and human thermal comfort levels reduce. This impact continued to be significant on subsequent warm days following the heatwave. This was also considered to lead to further impacts such as reduced pollution interception and uncontrolled stormwater runoff (30).

#### 3.3.6 The impact of animal and human health on ecosystem sustainability

In the eligible records, evidence was not found that the response to extreme weather events included measures to ensure that emergency responses included measures to reduce ecosystem stress and thus co-promote sustainable ecosystems, animal health and human health.

## 4 Discussion

Remarkably, despite the increasing importance of One Health approaches in the face of climate change impacts, true One Health approaches to the research of extreme weather events in eastern Australia are few in that there was very limited focus on the co-promotion of sustainable and integrated health of people, animals, and ecosystems. Our systematic search of peer-reviewed literature found only one study which considered aspects of all three domains (ecosystem, animal, and human health), and only two studies that made explicit recommendations from a One Health perspective. Due to the lack of studies addressing all three domains, we broadened the eligibility criteria to include studies in which two of ecosystem, animal and human health domains were included; however, this only increased the total number of eligible studies to ten, despite the frequency of extreme weather events in this region. This is an important outcome from this study, indicating that despite frequent extreme weather events in Australia that can cause impacts on ecosystem, animal, and human health, researchers rarely study the interdependencies between these domains.

Whilst there are too few studies to identify a clear pattern in the types of weather events that have been studied, research interest on specific types of weather events could reflect the frequency of types of extreme weather events in Australia, or possibly the duration or geographic extent of events. In the current review, the most studied type of extreme weather event was flooding followed by wildfire. As well as being the most frequent types of extreme weather event recorded by the National Emergency Management Agency (16), they are often also of relatively longer duration and more geographically widespread than other types of extreme weather events in the Australian context. Consistent with this, research on landslides and tidal waves was not found. Tidal waves are relatively infrequent – eastern Australia has experienced four tidal wave events since 2007, and none had significant morbidity, mortality or economic consequences (35), and there have also been relatively few landslides in eastern Australia, although in contrast to tidal waves, they have caused greater human morbidity and mortality (36). Cyclones are among the most economically costly extreme weather events, with sometimes major impacts on health. For example, Cyclone Yasi caused the evacuation of a major hospital, loss of human life, increased presentations of people to emergency departments, and was associated with an increase in the rate of antidepressant prescription post-disaster (14, 37–39). Despite this impact, cyclones were only investigated in one study (33).

As well as the frequency, duration and geographic extent, another feature that might generate research interest in research around extreme weather event is clear evidence of impact on people. The topics of research interest focused predominantly on outcomes on human health in two areas: infectious diseases (specifically, zoonoses and mosquito-borne diseases [MBDs]), and the psychological impact of extreme weather events in the context of the human-animal bond. Given that zoonoses involve at least one animal host as well as people, and that mosquito-borne pathogens have an ecosystem component due to the environment in which mosquitoes live, as well as often an animal host, this is not surprising – two domains of One Health would inherently be covered due to the nature of these infectious diseases. The link between the flooding events and mosquito abundance is also clear because the mosquito life-cycle requires occurs in aquatic ecosystems; therefore, the presence of floodwater can increase mosquito breeding site availability and thus the population and potentially, the force of infection of mosquito-borne viruses on animals and people (40–42). The reason for the focus on the psychological impact of extreme weather events is less clear; however, this might be due to changes in policies and plans in the wake of events such as Hurricane Katrina (New Orleans, USA in 2005) in which >1,000 people died and the difficulties of evacuation with pets was highlighted (43, 44). In a global review of the influence of companion animals in extreme weather events, owners’ made choices about evacuation that risked personal safety as well as the safety of emergency responders (45). A One Health approach to emergency management has been incorporated into emergency response systems in parts of the USA, Japan and Canada through the formation of specialist animal emergency response teams, however this system is lacking in Australia (46).

Recommendations for improved One Health policy, planning and responses to extreme weather events in Australia were only made in two studies, highlighting that inevitably (given the few studies), there is a gap in translating research findings into actionable One Health recommendations (27, 34). Whilst the focus of this review was research interest in One Health and extreme weather events, grey literature could offer valuable additional information that might increase our understanding of the scope of policy, planning and responses to extreme weather event management.

In comparison to research on emerging infectious disease and combatting antimicrobial resistance, it appears that the One Health impact and response to extreme weather events is an under-researched area. In addition to (zoonoses and MBDs integrating the direct physiological effects of extreme weather events on humans and animals and the damage to ecosystems); for example, due to pollution of waterways. Studies have shown that bushfire smoke is a known risk factor for adverse respiratory and cardiovascular effects in humans, livestock, companion animals, and wildlife (47, 48). Studies have also shown that bushfire and drought have altered water quality on the east coast of Australia (49).There were also no studies that investigated food security, considered a common One Health issue by the United States Centers for Disease Control and Prevention (50). International studies have shown links between weather events such as drought and reduced food security for both livestock and humans, however this was not explored by any studies included in this review (51).

## 5 Conclusion

Given the projected increase in frequency and intensity of extreme weather events, understanding their impacts on sustainable and integrated ecosystem, animal and human health will be critical to developing more effective promotion of One Health in this context. In Australia, studies were mainly limited to the most frequent extreme weather events (floods and wildfires), zoonoses and MBDs, and psychological impacts via the human-animal bond. There is a need to broaden this scope to ensure that policies, plans and responses in emergencies are comprehensive, feasible (including adaptable to local need), and effective.

## Author contributions

RW: Conceptualization, Data curation, Formal analysis, Investigation, Methodology, Software, Validation, Visualization, Writing – original draft, Writing – review & editing. VB: Conceptualization, Investigation, Methodology, Software, Supervision, Validation, Writing – original draft, Writing – review & editing. KR: Conceptualization, Investigation, Methodology, Project administration, Resources, Software, Supervision, Validation, Writing – original draft, Writing – review & editing.
