# Supplementary material for: Are we joining the One Health dots? A scoping review of research on the one health effects of extreme weather events in eastern Australia
Source: Front Vet Sci. 2024 Jul 29;11:1423501. doi: 10.3389/fvets.2024.1423501 (PMC11317470; doi:10.3389/fvets.2024.1423501)
Supplement: Supplementary file 1 [file Table_1.DOCX]

**Are we joining the One Health dots? A scoping review of research on the One Health effects of extreme weather events in eastern Australia**

**Rebecca Ward 1,5, Victoria Brookes 2,3†, Kazi Mizanur Rahman 4,5†***

1 Sydney Medical School, The University of Sydney, Camperdown, NSW, 2050, Australia

2 Sydney School of Veterinary Science, Faculty of Science, The University of Sydney, Camperdown, NSW, 2008, Australia

3 Sydney Institute for Infectious Diseases, Faculty of Medicine and Health, The University of Sydney, Camperdown, NSW, Australia

4 Faculty of Health Sciences and Medicine, Bond University, Robina, QLD 4226, Australia

5 University of Sydney, University Centre for Rural Health, Lismore, NSW 2480, Australia

**^*^Correspondence:**

Kazi Mizanur Rahman

[krahman@bond.edu.au](mailto:krahman@bond.edu.au)

†These authors contributed equally to this work and share last authorship

Supplementary Material

**Search terms used in each database**

**Medline (https://ovidsp.dc2.ovid.com)**

Ovid MEDLINE(R) ALL <1946 to June 16, 2023>

1 One Health/ 977

2 one health*.mp. 11400

3 one medicine*.mp. 266

4 planetary health*.mp. 964

5 ecohealth*.mp. 213

6 ((human* or "public health*" or "environmental health*") and (Animal* or wildlife* or livestock*)).mp. 2654581

7 ((human* or "public health" or "environmental health") and (ecosystem* or ecolog*)).mp. 103083

8 ((Animal* or wildlife* or livestock*) and (ecosystem* or ecolog*)).mp. 140658

9 1 or 2 or 3 or 4 or 5 or 6 or 7 or 8 2835496

10 exp natural disasters/ or cyclonic storms/ or droughts/ or floods/ or landslides/ or tornadoes/ or wildfires/ 25514

11 (natural disaster* or cyclon* or drought* or flood* or landslide* or tidal wave* or tornado* or wildfire* or extreme heat* or Extreme weather* or landslide* or heat wave*).mp. 74563

12 Extreme Weather/ or Extreme Cold Weather/ or Extreme Hot Weather/ 300

13 (extreme cold weather* or extreme hot weather*).mp. 211

14 (bushfire* or peat fire* or landscape fire*).mp. 609

15 10 or 11 or 12 or 13 or 14 79281

16 australian capital territory/ or new south wales/ or queensland/ or victoria/ 34152

17 (australian capital territory* or new south wales* or queensland* or victoria* or east* australia*).mp. 57579

18 16 or 17 57579

19 9 and 15 and 18 123

20 limit 19 to (english language and yr="2007 -Current") 106

**ProQuest (https://www.proquest.com)**

summary(("one health*" OR "one medicine" OR "planetary health*" OR "ecohealth*" OR ((human* OR "public health*" OR "environmental health*") AND (animal* OR wildlife* OR livestock*)) OR ((human* OR "public health" OR "environmental health") AND (ecosystem* OR ecolog*)) OR ((animal* OR wildlife* OR livestock*) AND (ecosystem* OR ecolog*)))) AND summary(("natural disaster*" OR cyclon* OR drought* OR flood* OR landslide* OR "tidal wave*" OR tornado* OR wildfire* OR drought* OR "extreme heat*" OR "extreme weather*" OR landslide* OR "heat wave*" OR "extreme cold weather*" OR "extreme hot weather*" OR bushfire* OR "peat fire*" OR "landscape fire*")) AND summary(("australian capital territory" OR "new south wales" OR victoria OR queensland OR "east* Australia*")) AND la.exact("English") AND pd(2007-2024)

**Scopus (https://www.scopus.com)**

(TITLE-ABS-KEY("one health*" OR "one medicine" OR "planetary health*" OR "ecohealth*" OR ( ( human* OR "public health*" OR "environmental health*" ) AND ( animal* OR wildlife* OR livestock* ) ) OR ( ( human* OR "public health" OR "environmental health" ) AND ( ecosystem* OR ecolog* ) ) OR ( ( animal* OR wildlife* OR livestock* ) AND ( ecosystem* OR ecolog* ) ))) AND (TITLE-ABS-KEY("natural disaster*"OR cyclon* OR drought* OR flood* OR landslide* OR "tidal wave*" OR tornado* OR wildfire* OR drought* OR "extreme heat*" OR "extreme weather*" OR landslide* OR "heat wave*" OR "extreme* weather*" OR "extreme cold weather*" OR "extreme hot weather*" OR bushfire* OR "peat fire*" OR "landscape fire*")) AND (TITLE-ABS-KEY("australian capital territory" OR "new south wales" OR victoria OR queensland OR "east* Australia*")) AND ( LIMIT-TO ( PUBYEAR,2023) OR LIMIT-TO ( PUBYEAR,2022) OR LIMIT-TO ( PUBYEAR,2021) OR LIMIT-TO ( PUBYEAR,2020) OR LIMIT-TO ( PUBYEAR,2019) OR LIMIT-TO ( PUBYEAR,2018) OR LIMIT-TO ( PUBYEAR,2017) OR LIMIT-TO ( PUBYEAR,2016) OR LIMIT-TO ( PUBYEAR,2015) OR LIMIT-TO ( PUBYEAR,2014) OR LIMIT-TO ( PUBYEAR,2013) OR LIMIT-TO ( PUBYEAR,2012) OR LIMIT-TO ( PUBYEAR,2011) OR LIMIT-TO ( PUBYEAR,2010) OR LIMIT-TO ( PUBYEAR,2009) OR LIMIT-TO ( PUBYEAR,2008) OR LIMIT-TO ( PUBYEAR,2007) ) AND ( LIMIT-TO ( LANGUAGE,"English" ) )

**Web of Science (https://www.webofscience.com)**

((TS=(( "one health*" OR "one medicine" OR "planetary health*" OR "ecohealth*" OR ( ( human* OR "public health*" OR "environmental health*" ) AND ( animal* OR wildlife* OR livestock* ) ) OR ( ( human* OR "public health" OR "environmental health" ) AND ( ecosystem* OR ecolog* ) ) OR ( ( animal* OR wildlife* OR livestock* ) AND ( ecosystem* OR ecolog* ) ) ) )) AND TS=("natural disaster*" OR cyclon* OR drought* OR flood* OR landslide* OR "tidal wave*" OR tornado* OR wildfire* OR drought* OR "extreme heat*" OR "extreme weather*" OR landslide* OR "heat wave*" OR "extreme* weather*" OR "extreme cold weather*" OR "extreme hot weather*" OR bushfire* OR "peat fire*" OR "landscape fire*")) AND TS=("australian capital territory" OR "new south wales" OR victoria OR queensland OR "east* Australia*") and 2007 or 2008 or 2009 or 2010 or 2011 or 2012 or 2013 or 2014 or 2015 or 2016 or 2017 or 2018 or 2019 or 2020 or 2021 or 2022 or 2023 (Publication Years) and English (Languages)

**Informit (https://search.informit.org/)**

[All Fields:"one health*" OR All Fields:"one medicine" OR All Fields:"planetary health*" OR All Fields:"ecohealth*" OR [[All Fields:( ( human* OR All Fields:"public health*" OR All Fields:"environmental health*" )] AND [All Fields:( animal* OR All Fields:wildlife* OR All Fields:livestock* ) )]] OR [[All Fields:( ( human* OR All Fields:"public health" OR All Fields:"environmental health" )] AND [All Fields:( ecosystem* OR All Fields:ecolog* ) )]] OR [[All Fields:( ( animal* OR All Fields:wildlife* OR All Fields:livestock* )] AND [All Fields:( ecosystem* OR All Fields:ecolog* ) )]]] AND [All Fields:"natural disaster*" OR cyclon* OR drought* OR flood* OR landslide* OR "tidal wave*" OR tornado* OR wildfire* OR “bushfire*” OR “peat fire*” OR “landscape fire*” OR "extreme heat*" OR “landslide*” OR “heat wave*” OR "extreme weather*" OR "extreme cold weather*" OR "extreme hot weather*" OR All Fields:"australian capital territory*" OR "new south wales*" OR Victoria* OR queensland* OR "east* Australia*" AND Limit To: Full Text AND Publication Date: (01/01/2007 TO 12/31/2023)

**CINAHL (https://web.p.ebscohost.com)**

(( MH "One Health Initiative") OR "One health*" ) OR "one medicine*" OR "planetary health*" OR "ecohealth*" OR ( "( human* or "public health" or "environmental health" ) AND ( ecosystem* or ecolog* )" ) OR ( "( Animal* or wildlife* or livestock* ) AND ( ecosystem* or ecolog* )" ) OR ( "( human* or "public health" or "environmental health" ) AND "( Animal* or wildlife* or livestock* ) )) AND ((MH "Natural Disasters") OR (MH "Wildfires")  OR (MH "Extreme Weather") OR ("natural disaster*" OR cyclon* OR drought* OR flood* OR landslide* OR "tidal wave*" OR tornado* OR wildfire* OR "extreme heat*" OR "extreme weather*" OR landslide* OR "heat wave*" OR "extreme cold weather*" OR "extreme hot weather*" OR bushfire* OR "peat fire*" OR "landscape fire*" ) AND ((MH "New South Wales") OR (MH "Queensland") OR (MH "Australian Capital Territory") OR (MH "Australian Capital Territory")  OR ("australian capital territory" OR "new south wales" OR victoria OR queensland OR "east* Australia*" OR australia*))

**Data extraction form**

| First author (last name) | (free text) |
| --- | --- |
| All disciplines involved in study | (free text) |
| One Health – all three, or pairing.   - Human health - Animal health - Ecosystem | (select all that apply) |
| Year published | (free text) |
| Journal | (free text) |
| Title | (free text) |
| Study population | (free text) |
| Study site | (free text) |
| Exposure of interest i.e. extreme weather events   - Flood - Bushfire - Drought - Heatwave - Landslide - Cyclone - Other | (select all that apply) |
| Outcome of interest | (free text) |
| Study period | (free text) |
| Data collection period | (free text) |
| Study design   - Qualitative - Quantitative - Mixed methods | (select all that apply) |
| Data collection tools   - Interview - Survey - Focus group - Case report - Other | (select all that apply) |
| Key findings related to the review question | (free text) |
| Recommendations from investigators related to the review question | (free text) |
| Study limitations | (free text) |
